# Pendelluft in hypoxemic patients resuming spontaneous breathing: proportional modes versus pressure support ventilation

**DOI:** 10.1186/s13613-023-01230-w

**Published:** 2023-12-20

**Authors:** Daniel H. Arellano, Roberto Brito, Caio C. A. Morais, Pablo Ruiz-Rudolph, Abraham I. J. Gajardo, Dannette V. Guiñez, Marioli T. Lazo, Ivan Ramirez, Verónica A. Rojas, María A. Cerda, Juan N. Medel, Victor Illanes, Nivia R. Estuardo, Alejandro R. Bruhn, Laurent J. Brochard, Marcelo B. P. Amato, Rodrigo A. Cornejo

**Affiliations:** 1 Departamento de Medicina, Unidad de Pacientes Críticos, Hospital Clínico Universidad de Chile, Dr. Carlos Lorca Tobar 999, 8380456 Santiago, Chile; 2 Departamento de Kinesiología, Facultad de Medicina, Universidad de Chile, Santiago, Chile; 3 Divisao de Pneumologia, Faculdade de Medicina, Instituto Do Coração, Hospital das Clinicas HCFMUSP, Universidade de São Paulo, São Paulo, Brazil; 4 Departamento de Fisioterapia, Universidade Federal de Pernambuco, Recife, Brazil; 5 Programa de Epidemiología, Facultad de Medicina, Instituto de Salud Poblacional, Universidad de Chile, Santiago, Chile; 6 Programa de Fisiopatología, Facultad de Medicina, Instituto de Ciencias Biomédicas, Universidad de Chile, Santiago, Chile; 7 Escuela de Kinesiología, Universidad Diego Portales, Santiago, Chile; 8 Departamento de Medicina Intensiva, Facultad de Medicina, Pontificia Universidad Católica de Chile, Santiago, Chile; 9 Center of Acute Respiratory Critical Illness (ARCI), Santiago, Chile; 10 Keenan Research Centre, Li Ka Shing Knowledge Institute, St. Michael’s Hospital, Unity Health Toronto, Toronto, Canada; 11 Interdepartmental Division of Critical Care Medicine, University of Toronto, Toronto, ON Canada

**Keywords:** Acute respiratory distress syndrome, Neurally-adjusted ventilatory assist, Proportional assist ventilation, Pressure support ventilation, Pendelluft

## Abstract

**Background:**

Internal redistribution of gas, referred to as pendelluft, is a new potential mechanism of effort-dependent lung injury. Neurally-adjusted ventilatory assist (NAVA) and proportional assist ventilation (PAV +) follow the patient’s respiratory effort and improve synchrony compared with pressure support ventilation (PSV). Whether these modes could prevent the development of pendelluft compared with PSV is unknown. We aimed to compare pendelluft magnitude during PAV + and NAVA versus PSV in patients with resolving acute respiratory distress syndrome (ARDS).

**Methods:**

Patients received either NAVA, PAV + , or PSV in a crossover trial for 20-min using comparable assistance levels after controlled ventilation (> 72 h). We assessed pendelluft (the percentage of lost volume from the non-dependent lung region displaced to the dependent region during inspiration), drive (as the delta esophageal swing of the first 100 ms [ΔP_es_ _100 ms_]) and inspiratory effort (as the esophageal pressure–time product per minute [PTP_min_]). We performed repeated measures analysis with post-hoc tests and mixed-effects models.

**Results:**

Twenty patients mechanically ventilated for 9 [5–14] days were monitored. Despite matching for a similar tidal volume, respiratory drive and inspiratory effort were slightly higher with NAVA and PAV + compared with PSV (ΔP_es 100 ms_ of –2.8 [−3.8–−1.9] cm H_2_O, −3.6 [−3.9–−2.4] cm H_2_O and −2.1 [−2.5–−1.1] cm H_2_O, respectively, *p* < 0.001 for both comparisons; PTP_min_ of 155 [118–209] cm H_2_O s/min, 197 [145–269] cm H_2_O s/min, and 134 [93–169] cm H_2_O s/min, respectively, *p* < 0.001 for both comparisons). Pendelluft magnitude was higher in NAVA (12 ± 7%) and PAV + (13 ± 7%) compared with PSV (8 ± 6%), *p* < 0.001. Pendelluft magnitude was strongly associated with respiratory drive (β = -2.771, p-value < 0.001) and inspiratory effort (*β* = 0.026, *p*  < 0.001), independent of the ventilatory mode. A higher magnitude of pendelluft in proportional modes compared with PSV existed after adjusting for PTP_min_ (*β* = 2.606, *p* = 0.010 for NAVA, and β = 3.360, *p* = 0.004 for PAV +), and only for PAV + when adjusted for respiratory drive (β = 2.643, *p* = 0.009 for PAV +).

**Conclusions:**

Pendelluft magnitude is associated with respiratory drive and inspiratory effort. Proportional modes do not prevent its occurrence in resolving ARDS compared with PSV.

**Supplementary Information:**

The online version contains supplementary material available at 10.1186/s13613-023-01230-w.

## Background

One of the main challenges in patients with acute respiratory distress syndrome (ARDS) is the transition from controlled to partial support ventilation due to the potential risks of spontaneous breathing balanced against the risks of controlled ventilation. On one hand, the inactivity of the diaphragm may promote early diaphragmatic dysfunction [1, 2]. On the other hand, spontaneous breathing has been associated to better gas distribution, ventilation-perfusion matching, cardiac performance, clearance of secretions and respiratory muscle function [3]. However, vigorous spontaneous breathing may induce the mechanisms of effort dependent lung injury, including intrapulmonary pendelluft and, thereby, may complicate the ventilator liberation process [4, 5]. Pendelluft can be an injurious lung inflation pattern that often amplifies regional stress, strain and tidal recruitment at dependent regions during strong inspiratory efforts [4, 5]. We previously showed that high magnitude pendelluft could be a potential determinant of inflammatory response related to inspiratory efforts in ARDS [6].

In this setting, proportional modes of ventilation could be an interesting alternative. Neurally adjusted ventilatory assist (NAVA) and proportional assist ventilation plus (PAV +) are forms of partial ventilatory support that can decrease ventilator patient-asynchrony and enhance the patient’s control mechanisms against both lung overdistention and ventilator overassistance, thereby protecting the lungs [7, 8].

The evidence supporting proportional modes comes mainly from physiological studies in heterogeneous groups of patients [7, 8]. Beneficial effects of NAVA have been found in reducing the duration of ventilation but not mortality [9]. It is unclear whether a protective role may be extrapolated to moderate-severe ARDS patients recovering spontaneous breathing. This group of patients is of particular interest since their breathing pattern can result in pendelluft [4, 5].

The better inspiratory synchrony and match with the patient’s respiratory effort during proportional modes could prevent the development of pendelluft. However, the slower increase in airway pressure during the beginning of inspiration in proportional modes, compared with pressure support ventilation (PSV) [10, 11], may lead to an increase in intrapulmonary pendelluft, especially in the presence of high respiratory drive.

Hence, we aimed to compare the effects of NAVA and PAV + versus PSV on pendelluft magnitude, and to analyze the associations between pendelluft with respiratory drive and inspiratory effort in ARDS patients recovering spontaneous breathing.

## Methods

### Study population

We included patients who had moderate–severe ARDS in the early phase and received controlled ventilation for over 72 h, in whom the attending physician had decided the transition from controlled to partial ventilatory support 24 h before the spontaneous study onset and were under moderate-light sedation (Richmond Agitation-Sedation Scale −2 to −3). The patients maintained spontaneous breathing under partial support ventilation (assisted-PCV and/or BILEVEL) until the spontaneous modes trial started. This population was chosen to enrich the study sample with patients more likely to present pendelluft during assisted/spontaneous ventilation. The study was approved by the Institutional Ethics Committee (N.027/2016). Informed consent was obtained from the patients’ relatives. Patients younger than 18 years old, pregnant, with contraindications to place the electrical impedance tomography (EIT) system or nasogastric tube, central nervous system injury, chronic neuromuscular disease, new sepsis, moderate-severe metabolic acidosis [12], obstructive lung diseases or intrinsic end-expiratory pressure (PEEP) ≥ 3 cm H_2_O, and respiratory or hemodynamic instability [13], were excluded. Duration of mechanical ventilation was not an exclusion criterion. Further details are described in the Additional file 1.

### Study protocol

This was a clinical–physiological crossover study and we compared pendelluft magnitude in NAVA and PAV + versus PSV. An individualized level of positive end-expiratory pressure (PEEP) was set considering the lowest combination of collapse and overdistension according to EIT monitoring [14, 15].

We adjusted the level of assistance in each mode intending to keep a similar tidal volume (V_T_ 6–8 ml/kg of predicted body weight [PBW]) and esophageal swing (< 15 cm H_2_O), and adequate adaptation of the patient. Subsequently, NAVA, PSV, and PAV + were randomly applied in a crossover trial for 20 min each. We provided a 10 min-washout period between modes using assisted pressure-controlled ventilation (PCV). Further details of the titration strategy are described in the Additional file 1.

**Fig. 1:**
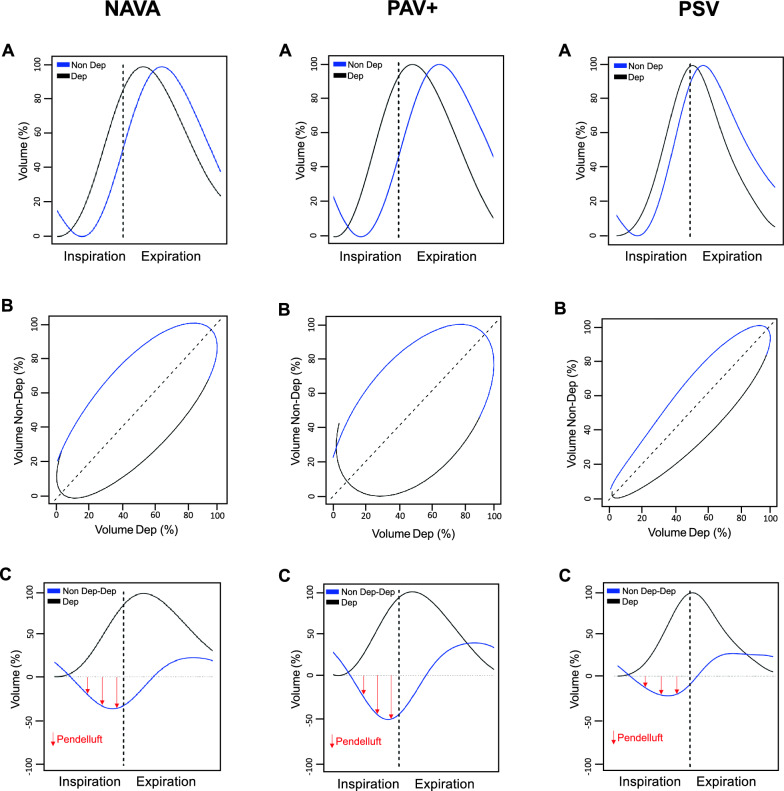
Illustrations of pendelluft magnitude assessment in NAVA, PAV + and PSV modes from a representative patient (#8). Panel **A** represents the change of lung volume (0–100%) during inspiration and expiration for non-dependent (blue line) and dependent lung regions (black line). The dashed vertical line delimits the inspiration time. In these representative ventilatory cycles with pendelluft, non-dependent region loses volume at the early stage on inspiration while dependent region starts inflation, producing that the wave of the non-dependent region would lag behind the dependent region. The phase angle visualization (panel **B**) allows to evidence the lost volume from non-dependent region with concomitant gain volume in dependent region during inspiration and not just inflation delay. Panel **C** illustrates the volume displacement between ventral and dorsal regions, which is calculated as the average difference between non-dependent and dependent volume (%) throughout the inspiration (solid blue line in panel **C**). Note that the greater the negative swing the greater the magnitude of pendelluft, as indicated by the red arrows

We assessed pendelluft, respiratory drive and inspiratory effort in the same ventilatory cycles to analyze the direct association between these variables. Occlusion maneuvers were not applied. We used a method based on EIT (Enlight 1800, Timpel^®^) to quantify pendelluft as the percentage of lost volume from non-dependent lung region displaced to the dependent region during inspiration [6]. This method which is summarized in Fig. 1 does not require a control breath as comparator. In each ventilatory mode, we calculated the average magnitude of pendelluft and determined the frequency of ventilatory cycles with pendelluft magnitude exceeding 15%, 20% and 25%, as these thresholds have been associated with an increase in inflammatory biomarkers [6]. Airway pressure (P_aw_), esophageal pressure (P_es_), gastric pressure (P_g_), transpulmonary pressure (P_L_) and transdiaphragmatic pressure (P_di_) and flow were recorded using pressure transducers and a pneumotachometer (FluxMed MBMED^®^). The correct position of the esophageal catheter (Neurovent Research Inc^®^, Canada) was confirmed as described previously [16]. P_L_ was calculated as the difference between P_aw_ and P_es_, and P_di_, as the difference between P_g_ and P_es_. We used dynamic ΔP_L_, ΔP_es_, and ΔP_di_ in reference to the end-expiratory value. Ventilatory ratio was calculated as a surrogate for pulmonary dead space [17]. To estimate respiratory drive, we analyzed the delta esophageal swing of the first 100 ms (ΔP_es 100 ms_) as proxy of airway occlusion pressure (P_01_), knowing that the inspiratory trigger delay reported for these three spontaneous modes is higher than 100 ms [18]. Additional indices of respiratory drive (dP_di_/dt and dP_es_/dt) were also obtained (Additional file 1). To assess the inspiratory effort, we calculated the pressure–time product per minute from consecutive breaths as the area subtended between P_es_ and the chest wall recoil pressure during inspiration multiplied by respiratory rate (PTP_min_) [19]. To evaluate the early inspiratory workload, we analyzed the PTP of the first 300 ms from the onset of inspiration (PTP_300ms_). Gas-exchange analysis was performed after each mode. Respiratory mechanics during PAV + and dorsal fraction of ventilation (i.e., the ratio between tidal volume in the dependent region and total tidal volume multiplied by 100) in cycles with high pendelluft magnitude (defined as > 20–25%) with respect to low pendelluft magnitude (defined as 10–15%), were included in the Additional file 1.

## Statistical analysis

Considering the physiological design of the study, a formal sample size calculation was not initially conducted. However, in line with previous research on the field [20, 21], we intended to recruit a convenience sample consisting of 20 patients. Our preliminary data estimating pendelluft and standard deviation from the first 5 patients also suggested a sample size of 18 patients to find an effect size of 0.7 on pendelluft magnitude, with 0.5 correlation among repeated measures, 5% significance level and 80% power.

Shapiro–Wilks’ test was used to assess normality. Data are expressed as mean ± standard deviation or median [interquartile range]. Repeated measures ANOVA or Friedman test, followed by Dunnett ‘s or Dunn's post-hoc test, were performed to compare variables from NAVA and PAV + versus PSV. Linear mixed-effects models (LMM) with patients as random intercepts were performed to associate pendelluft magnitude with respiratory drive, inspiratory effort variables or ventilatory modes. In addition, we assessed ΔP_es 100 ms_ and PTP_min_ as potential predictors of pendelluft magnitude, using repeated measures analysis with LMM to estimate a marginal R^2^ (R^2^_LMM_) applying constant slopes and random intercepts of the unadjusted and adjusted models (including the ventilatory modes). Wilcoxon signed-rank test was used to compare the changes in PTP_min_ between titration and trial in each mode. Analyses were performed in Stata v 14.0 and GraphPad Prism v 9.0. Additional analyses for respiratory mechanics during PAV + and dorsal fraction of ventilation in ventilatory cycles with low and high magnitude of pendelluft, were included in the Additional file 1.

## Results

Twenty patients were included in the study (age 61 [50–67] years, body mass index 30 [23–33] kg/m^2^, 6 females). The cause of ARDS was pulmonary sepsis in 13, extrapulmonary sepsis in 5, and other non-septic inflammatory diseases in 2 patients. Fifteen patients had received neuromuscular blocking agents (11 by prolonged continuous infusion and 4 by intermittent bolus injection). Five patients required prolonged prone position ventilation and two were subjected to repeated abdominal surgeries before spontaneous breathing onset. At the study entry, mechanical ventilation time was 9 [5–14] days, and PaO_2_:FiO_2_ ratio 275 ± 46 mmHg and PaCO_2_ 39 ± 5 mmHg. Respiratory system and chest wall compliances were 38 [30–47] and 143 [110–157] mL/cm H_2_O, respectively. PEEP level was 10 [7–12] cm H_2_O. Esophageal/gastric-related data were obtained from 19 of the 20 patients (Further clinical and ventilatory details are provided in Additional file 1: Table S1 and S2).

## Assistance titration

The values of target assistances during partial support ventilation modes were 10 [5–10] cm H_2_O in PSV, 1.0 [1.0–1.0] cmH_2_O/μV in NAVA and 50 [40–59] % gain in PAV + , which during the initial titration produced comparable V_T_ (~ 7.4 mL/Kg PBW), esophageal pressure swing (~ 7.7 cmH_2_O) and PTP_min_ (~ 135 cmH_2_O s/min). See Additional file 1: Table S3. Individual data of assistance during the titration period and the trial are available in Additional file 1: Table S2 and Figure S1.

## Crossover trial

Table 1 shows the comparison between NAVA and PAV + versus PSV on the main respiratory variables, using the settings determined during the titration phase. Respiratory rate (RR), V_T_, minute volume ventilation and oxygen exchange were similar in the three modes. Although the rest of respiratory variables were comparable during assistance titration, at the time of the crossover trial ΔP_es_ and ΔP_di_ were higher in PAV + , while dynamic ΔP_L_ was higher in NAVA,  with respect to PSV. Compared with PSV, peak Paw was higher in NAVA and lower in PAV + . In addition, a higher ventilatory ratio was observed in NAVA compared to PSV (Table 1). Quasi-static values of airway driving pressure and transpulmonary driving pressure during PAV + were 12.4 [10.5–16.6] and 8.6 [7.1–10.2] cm H_2_O, respectively (Additional file 1: Figure S2).

**Table 1:** Respiratory variables during crossover trial

	NAVA	PAV +	PSV	p-value
Tidal volume, ml/kg PBW, median [IQR]	7.8 [7.4–8.9]	7.9 [7.2 – 8.8]	7.7 [7.2–9.3]	0.8589
Respiratory rate, bpm, mean ± SD	24.9 ± 7	24.9 ± 7	22.3 ± 6	0.0604
Volume minute ventilation, mean ± SD	11.6 ± 2.6	11.2 ± 2.8	10.4 ± 3.1	0.2312
Peak P_aw_, cm H_2_O, mean ± SD	21.1 ± 4.9*	16.7 ± 4.0*	18.7 ± 3.9	< 0.0001
ΔP_es_, cm H_2_O, mean ± SD	−8.4 ± 3.1	−10.9 ± 3.9*	−7.3 ± 3.4	0.0004
ΔP_L_, cm H_2_O, median [IQR]	17.8 [16.4–24.8]*	17.5 [14.7–21.1]	15.4 [14.4–17.3]	0.0083
ΔP_di_, cm H_2_O, median [IQR]	7.5 [5.3–9.8]	9.2 [7.6–11.4]*	7.3 [4.6–9.1]	< 0.0001
PaO_2,_ mmHg, median [IQR]	75.7 [72.7–83.1]	80.9 [72.9–97.3]	80.7 [74.3–88.7]	0.3867
PaO_2_:FiO_2,_ mean ± SD	276 ± 80	284 ± 74	285 ± 68	0.6730
PaCO_2_, mmHg, mean ± SD	39.2 ± 5.2	39.5 ± 5.2	39.5 ± 4.9	0.7729
pH, median [IQR]	7.4 [7.4–7.5]	7.4 [7.4–7.5]	7.4 [7.4–7.5]	0.4813
Ventilatory ratio	2.1 ± 0.7*	2.0 ± 0.5	1.8 ± 0.4	0.0310

p-value: significance of repeated measures ANOVA or Friedman test

*NAVA* neurally-adjust ventilatory assist, *PAV* proportional assist ventilation, *PSV* pressure support ventilation, *PBW* predicted body weight, *IQR* interquartile range, *SD* standard deviation, *P*_*aw*_ airway pressure, *ΔP*_*es*_ esophageal pressure swing, *ΔP*_*L*_ dynamic transpulmonary pressure, *ΔP*_*di*_ transdiaphragmatic pressure, *PaO*_*2*_ arterial pressure of oxygen, *FiO*_*2,*_ inspired fraction of oxygen, PaCO_2_ arterial pressure of carbon dioxide; Ventilatory ratio is a unit less index calculated as (minute ventilation in ml/min x PaCO_2_) / (Ideal body weight × 100 × 37.5)

^*^p < 0,05 compared with PSV

**Fig. 2:**
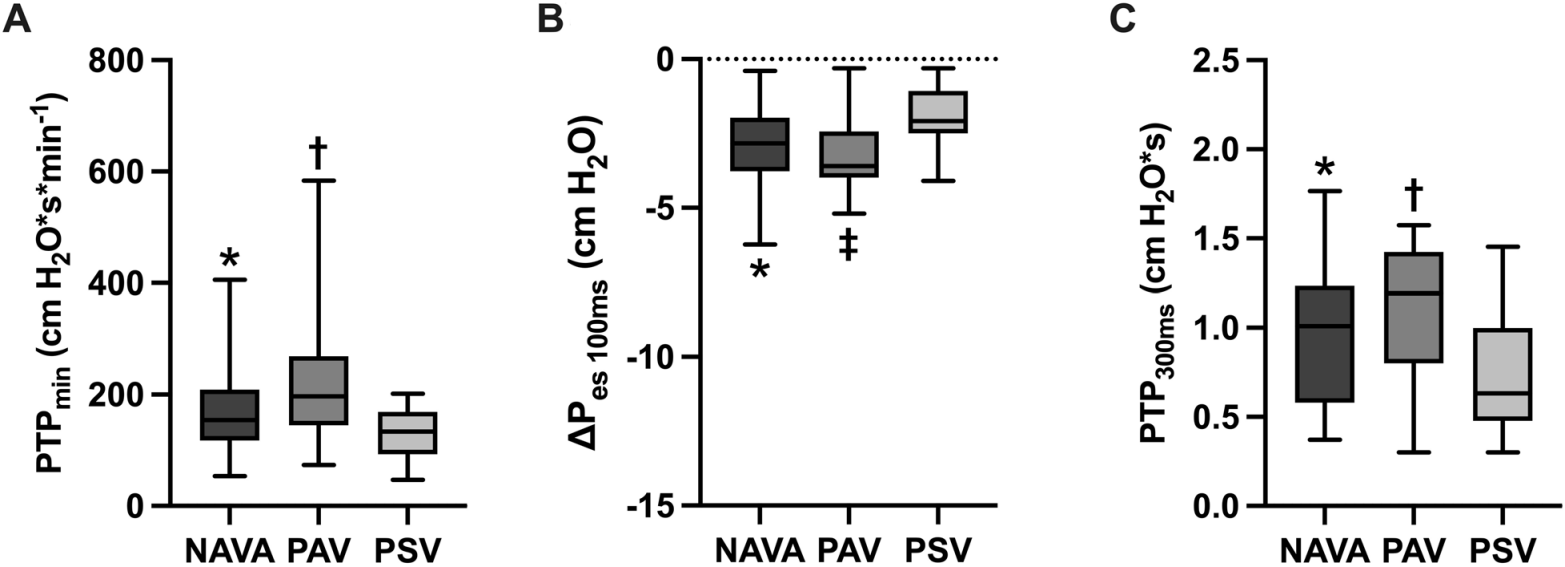
Respiratory drive and inspiratory effort variables during partial support ventilation modes. Compared with PSV, patients during proportional modes (NAVA and PAV +) presented a higher pressure–time product [PTP] per minute (panel **A**), a higher delta P_es_ at the first 100 ms from the onset of inspiration [ΔP_es 100 ms_] (panel **B**), and a higher PTP during the first 300 ms [PTP_300ms_] (panel **C**) (*p < 0,05; ^†^*p* < 0,001; and, ^‡^*p* < 0,0001 all compared with PSV)

The representative variables of respiratory drive (ΔP_es 100 ms_) and early inspiratory workload (PTP_300ms_) exhibited significantly higher values in proportional modes compared with PSV (Fig. 2). Among the additional indices of respiratory drive, only dP_di_/dt was higher in NAVA compared with PSV (See Additional file 1: Figure S3). The PTP_min_ was also slightly higher in both NAVA and PAV + with respect to PSV during the trial (Fig. 2). Indeed, there was an unexpected increase in PTP_min_ in proportional modes between the assistance titration period and the crossover trial (up to 155 [118–209] cm H_2_O s/min in NAVA, and up to 197 [145–269] cm H_2_O s/min in PAV + , *p* < 0.001 for both modes), but not in PSV (whose value was 134 [93–169] cm H_2_O s/min in trial, *p* = 0.1415).

## Pendelluft magnitude and determinants

**Fig. 3:**
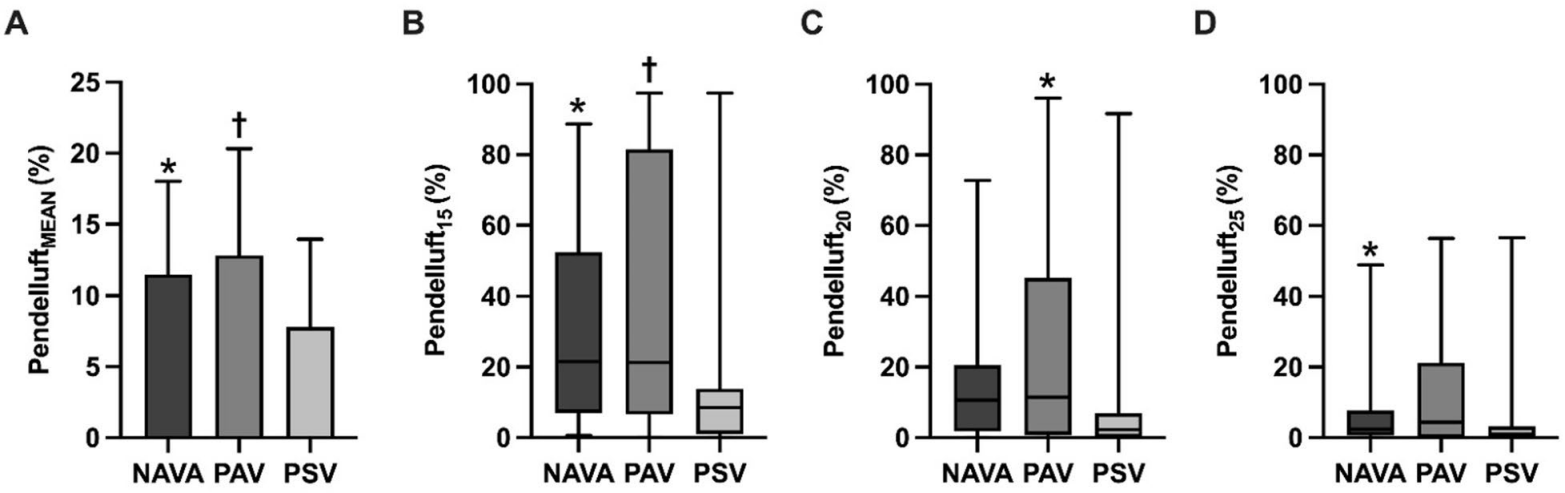
Comparison of pendelluft magnitude and the frequency of pendelluft with magnitudes above specific thresholds between proportional modes and pressure support ventilation. Compared with PSV, patients during NAVA and PAV + exhibited a higher mean pendelluft magnitude (Panel **A**). When analyzing pendelluft at different magnitude cutoffs, both NAVA and PSV + had a higher frequency at 15% magnitude (Panel **B**), but only PAV + was higher at 20% magnitude and NAVA at 25% magnitude compared with PSV (Panel **C** and **D**, respectively) (*p < 0,05; and, ^†^*p* < 0,001 all compared with PSV)

The mean pendelluft magnitude was significantly higher in NAVA and PAV + when compared with PSV (Fig. 3). Similarly, the frequency of ventilatory cycles with pendelluft magnitude above 15% threshold was consistently higher in proportional modes with respect to PSV. At higher cut-off points of pendelluft magnitude, both PAV + and NAVA showed a higher frequency of ventilatory cycles with magnitude exceeding 20% and 25%, when compared with PSV (Fig. 3).

Taking all modes and patients together, the magnitude of pendelluft was found to be associated with respiratory drive (ΔP_es 100 ms_, dP_di_/dt and dP_es_/dt), inspiratory effort (PTP_min_ and ΔP_di_,), and early inspiratory workload (PTP_300ms_), independently of the effect of ventilatory mode (*p* < 0.001 for all the associations; see Table 2). In adjusted models, as examples, for every 1.0 cm H_2_O increase in ΔP_es 100 ms_, 100-units increase in PTP_min_, 3 cm H_2_O increase in ΔP_di_, and 0.35-units increase in PTP _300 ms_, an additional 1.7 to 2.0% of lost volume from non-dependent lung region is displaced to the dependent region during inspiration (pendelluft).

**Table 2:** Effect of inspiratory effort and respiratory drive on pendelluft and the association between proportional modes and pendelluft compared to PSV

Independent variables	Pendelluft _MEAN_
	β (p-value)
Indices of respiratory drive	
ΔP_es 100 ms_	
Unadjusted	−2.771 (p < 0.001)
Adjusted by mode	−1.977 (p = 0.001)
dP_es_/dt	
Unadjusted	−0.550 (p < 0.001)
Adjusted by mode	−0.373 (p = 0.003)
dP_di_/dt	
Unadjusted	0.665 (p < 0.001)
Adjusted by mode	0.466 (p = 0.002)
Inspiratory effort variables	
PTP_min_	
Unadjusted	0.026 (p < 0.001)
Adjusted by ventilatory mode	0.017 (p = 0.003)
PTP_300ms_	
Unadjusted	9.019 (p < 0.001)
Adjusted by ventilatory mode	5.508 (p = 0.010)
ΔP_di_	
Unadjusted	0.857 (p < 0.001)
Adjusted by ventilatory mode	0.641 (p = 0.001)
Ventilatory Modes	
NAVA	
Unadjusted	3.707 (p = 0.001)
Adjusted by ΔP_es 100 ms_	1.529 (p = 0.172)
Adjusted by PTP_min_	2.606 (p = 0.010)
PAV +	
Unadjusted	5.027 (p < 0.001)
Adjusted by ΔP_es 100 ms_	2.643 (p = 0.033)
Adjusted by PTP_min_	3.360 (p = 0.004)

β (p-value): Regression coefficient and p-value of each mixed-effects model

*ΔP*_*es 100 ms*_ esophageal pressure swing of the first 100 ms from the onset of inspiration, *dP*_*es*_*/dt* change over time of esophageal pressure during the inspiratory phase, *dP*_*di*_*/dt* change over time of transdiaphragmatic pressure during the inspiratory phase, *PTP*_*min*_ pressure–time product per minute, *ΔP*_*di*_ transdiaphragmatic pressure, *PTP*_*300ms,*_ pressure time product of the first 300 ms from the onset of inspiration: *NAVA* neurally-adjusted ventilatory assist, *PAV + * proportional assist ventilation, *PSV* pressure support ventilation

In addition, there was an association between proportional modes and pendelluft with respect to PSV (Table 2). After including ΔP_es 100 ms_ or PTP_min_ (representative variables of drive and effort, respectively) into the model, the strength of this association was reduced. However, even after adjusting for PTP_min_, a higher magnitude of pendelluft in proportional modes compared with PSV persisted (*p* = 0.01 for NAVA and *p* = 0.04 for PAV +). Only PAV + maintained the association with pendelluft when adjusted for ΔP_es 100 ms_ (*p* = 0.033), Table 2.

**Fig. 4:**
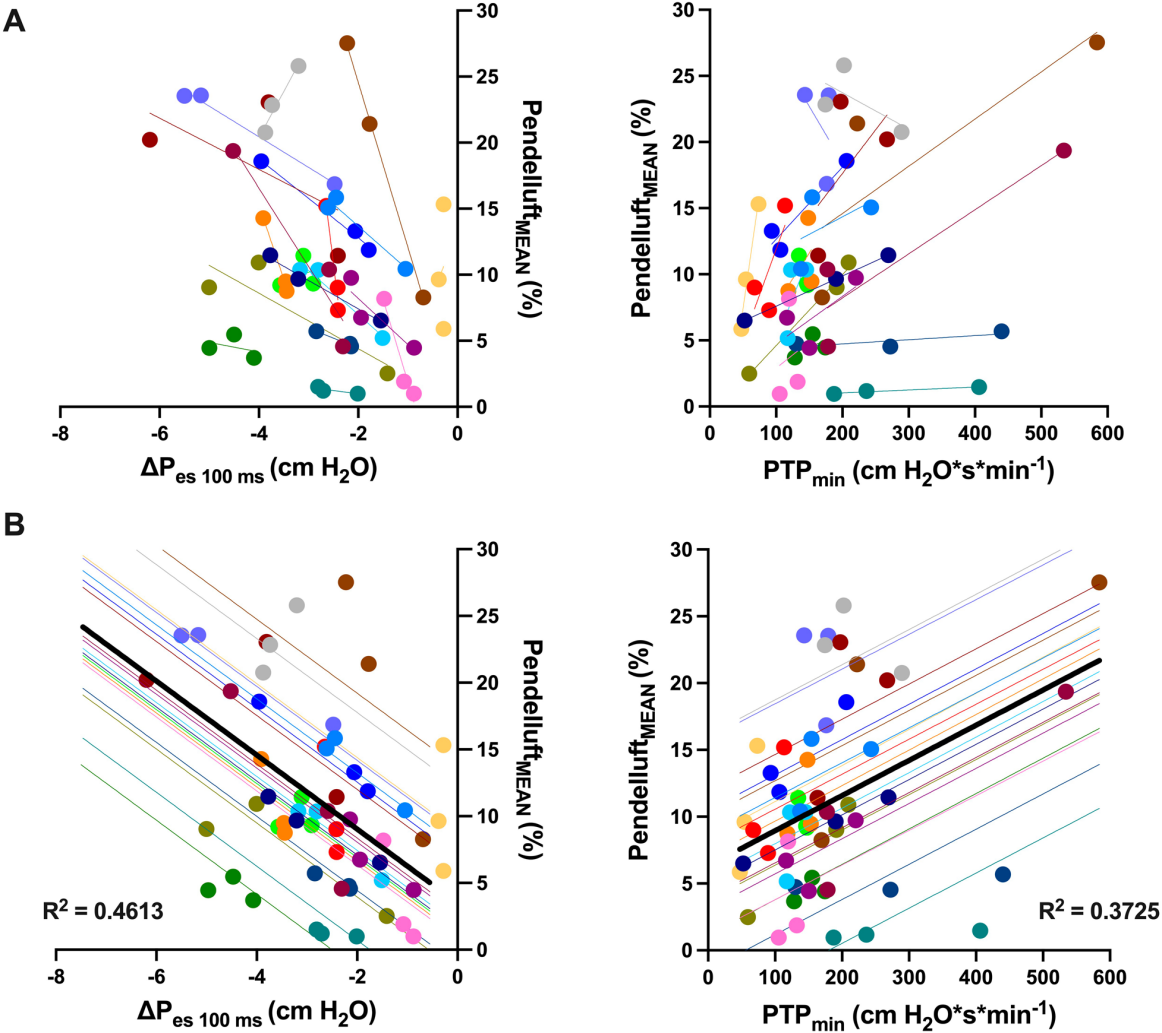
Correlations between pendelluft magnitude and ΔP_es 100 ms_ or PTP_min_. Each color represents a subject. Solid circles represent the average of ΔP_es 100 ms_ or PTP_min,_ and pendelluft magnitude from each patient in each mode. In panel **A**, solid lines represent the slope of the simple regressions of pendelluft with ΔP_es 100 ms_ (left) or PTP_min,_ (right) by patient. In panel **B**, solid lines represent the slope of the unadjusted regressions from repeated measures analysis with linear mixed-effects models for each patient. Black solid line corresponds to the regression model representative of all patients

With the exception of two patients, all participants demonstrated direct associations in their simple linear regressions between ΔP_es 100 ms_ or PTP_min_ and pendelluft including the three modes (Fig. 4A). In the LMM, the R^2^_LMM_ was 0.4613 (unadjusted) and 0.5084 (adjusted by mode) between ΔP_es 100 ms_ and pendelluft mean. On the other hand, the R^2^_LMM_ was 0.3725 (unadjusted) and 0.4960 (adjusted by mode) between PTP_min_ and pendelluft mean. The results of the unadjusted models were graphically shown (Fig. 4B).

**Fig. 5:**
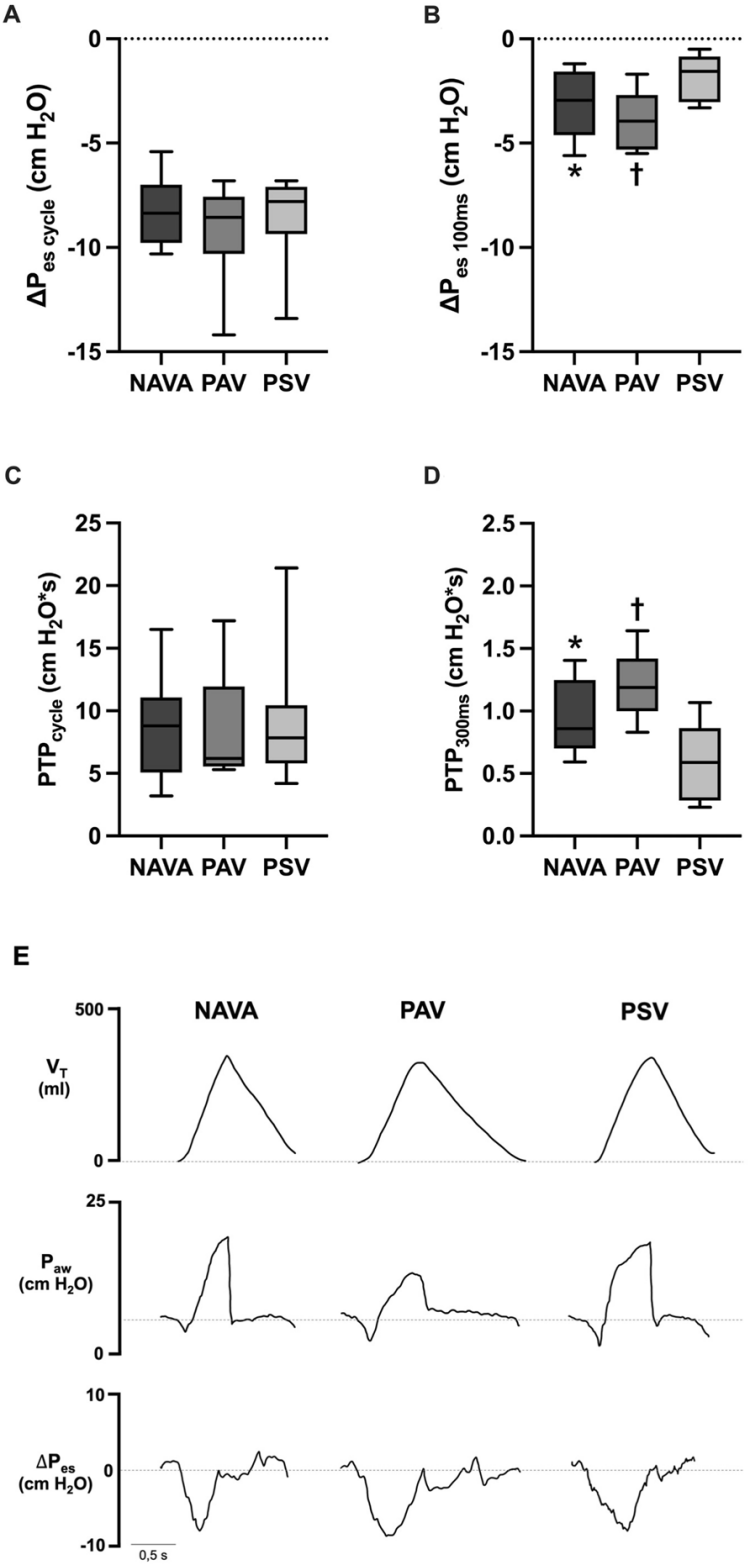
Drive and effort at the beginning of inspiration in representative ventilatory cycles with similar esophageal swing from selected patients in NAVA, PAV + and PSV. Ten representative ventilatory cycles in each ventilatory mode (NAVA, PAV + and PSV) with similar magnitude of esophageal swing from 5 selected patients were analyzed. No differences were observed in esophageal swings and pressure–time product [PTP] per cycle between proportional modes and PSV (**A** and **C**, respectively). By contrast, delta P_es_ during the first 100 ms [ΔP_es 100 ms_] (**B**) and PTP during the first 300 ms [PTP_300ms_] (**D**) were significantly higher in proportional modes than in PSV. Representative ventilatory tracings from patient #8 are shown in letter **E**. The values of V_T_, ΔP_es_ per cycle, ΔP_es 100 ms_ and PTP_300ms_ during NAVA, PAV + and PSV are 347, 339 and 348 ml; −8.8, −9,2 and −8.8 cm H_2_O; −4.3, −5.3 and −1.7 cm H_2_O, and 1.26, 1.38 and 0.85 cm H_2_O*s, respectively (*p < 0,05; and, ^†^*p* < 0,001 all compared with PSV)

To investigate potential reasons for the higher magnitude of pendelluft in proportional modes compared with PSV after controlling by PTP_min_, we analyzed 10 ventilatory cycles from cases in which the esophageal swing per cycle was matched in across all modes. Despite a similar ΔP_es_ and PTP per cycle, we found that NAVA and PAV + presented slightly higher ΔP_es 100 ms_ and PTP_300ms_ with respect to PSV (Fig. 5). Representative ventilatory tracings are shown in Fig. 5E.

## Pendelluft and regional ventilation

The dorsal fraction of ventilation was slightly higher in cycles with high pendelluft magnitude compared with cycles with low pendelluft magnitude at similar V_T_ in the three modes (Additional file 1: Figure S4), although a further increase was observed in PAV + (Additional file 1: Figure S5). Furthermore, the dorsal fraction of ventilation was also higher in PAV + , compared with NAVA and PSV, both in cycles with low and high magnitude of pendelluft (Additional file 1: Table S5).

## Discussion

In ARDS patients recovering from the acute phase, previously receiving controlled ventilation for at least 72 h, NAVA and PAV + did not protect against pendelluft compared with PSV. The higher pendelluft magnitude in proportional modes in the study was related to an increase in inspiratory effort and higher respiratory drive in both NAVA and PAV + despite a similar tidal volume and minute ventilation.

As pendelluft is directly related to the inspiratory effort [22], the higher pendelluft magnitude in proportional modes during the trial was partially explained by the higher PTP_min_. Interestingly, the association between proportional modes and pendelluft was maintained also adjusting for concurrent PTP_min_ in a regression model. Additionally, to investigate potential underlying mechanisms connecting the proportional modes with pendelluft, we analyzed cycles from a subsample of patients with similar esophageal swings and PTP per cycle in the three modes. This exploratory analysis suggests a higher respiratory drive and higher inspiratory workload at the early stage of inspiration during NAVA and PAV + , possibly attributed to a slower pressurization rate compared to PSV [10, 11, 23, 24] (Fig. 4E). In bench studies, irrespective of respiratory mechanics and gain, PAV + provides a P_aw_ approximately 25% lower than expected, being this under-assistance greater at the beginning of the inspiration [11]. Also, a delay on elastic and resistive unloading has been described during PAV [25]. In addition, NAVA may increase the tidal ventilation of the dependent lung region compared with PSV at the same pressure in patients with acute lung injury, even at high assistance [26]. All the aforementioned factors suggest that work of breathing may not be always well supported at the beginning of inspiration during proportional modes, which could promote intrapulmonary dyssynchrony (pendelluft) especially in ARDS patients with high respiratory drive.

Clinicians should be aware that during different partial support ventilation modes a good matching in tidal volume does not guarantee the same level of inspiratory effort and/or respiratory drive [27, 28]. Indeed, despite a similar tidal volume and minute ventilation, the patients with proportional modes exhibited higher respiratory drive and higher inspiratory effort. Although there was not overassistance in PSV [29], the lack of systematic comparisons at the same drive and effort do not allow to conclude an intrinsic effect of proportional modes on pendelluft phenomenon, but rather mediated through these variables. The moderate correlation obtained between ΔP_es 100 ms_ or PTP_min_ and pendelluft further supports this interpretation.

Pendelluft may generate overdistension of dorsal regions, which is more likely to occur in cycles with high pendelluft magnitude. In addition, the intrapulmonary gas volume displacement does not contribute to gas exchange. Both of these phenomena might cause a transitory and modest increase in CO_2_ levels, enough to raise the wasted work of breathing. Although speculative, the higher ventilatory ratio in NAVA (and its trend in PAV +) compared with PSV could be attributed to an underlying vicious circle of increased respiratory workload, pendelluft and ventilatory inefficiency [21].

The small increase in dorsal fraction of ventilation observed in ventilatory cycles with high pendelluft magnitude is similar to the found in other physiological study [21], but of uncertain significance. To the best of our knowledge, only a few studies have explored the potential clinical impact of pendelluft [21, 30, 31]. In one of these studies, pendelluft was associated with a longer duration of mechanical ventilation among ICU patients with PaO_2_/FiO_2_ ratio below 200 mmHg. Interestingly, the authors observed this association despite dorsal fraction of ventilation was not different between patients with and without pendelluft [30]. Whether this outcome is related with pendelluft itself or effort (or other confounding variables) is unknown.

The main contribution of this study is the assessment of proportional modes in a specific stage of the ARDS with focus on pendelluft magnitude. Among the methodological strengths are a well-defined population, titration of individualized levels of PEEP and assistance, and the objective measurements of inspiratory effort using a physiological state-of-the-art approach. However, our findings must be interpreted with caution due to several limitations such as: (1) being a clinical–physiological study of limited size; (2) the limited time for assessments on each spontaneous mode during the titration period and the crossover trial; (3) the lack of other measurements to estimate effort and drive using inspiratory and expiratory holds; (4) the exploratory nature of the inspiratory effort analysis at the beginning of inspiration matching esophageal swing and PTP per cycle; (5) EA_di_ signal was available only in NAVA, (6) the lack of a diaphragmatic dysfunction assessment which might alter the performance of proportional modes, and (7) specific and more reliable indices of ventilatory inefficiency were not available.

We do not believe these findings are a signal that favors PSV over NAVA and PAV + but highlight the necessity of respiratory monitoring of drive and effort during spontaneous modes in ARDS patients recovering spontaneous breathing. NAVA and PAV + are designed to adjust the level of assistance proportionally to the patient’s effort. Our findings further reinforce the need to avoid under-assistance. Although NAVA and PAV + were associated with a higher magnitude of pendelluft, the magnitudes reported are of unknown clinical significance. Further studies are needed to establish the clinical impact of these findings.

## Conclusions

NAVA and PAV + did not protect against pendelluft compared with PSV. The magnitude of pendelluft is directly associated with respiratory drive and inspiratory effort and could increase during proportional modes in ARDS patients recovering spontaneous breathing when compared with PSV. The most likely explanation for those findings is the transient under-assistance of proportional modes during early inspiration.

### Supplementary Information

**Additional file 1: Table S1.** Demographic and clinical characteristics of the study population. **Table S2.** Patients’ data at the study entry and the individualized levels of PEEP and comparable levels of assistance in NAVA, PAV + and PSV during the trial. **Table S3.** Comparison of tidal volume, esophageal swing and pressure–time product per minute at the titration period between NAVA, PAV + and PSV. **Table S4.** Individual differences of the respiratory variables during crossover trial, using PS.as reference value. **Table S5.** Comparison of tidal volume and dorsal fraction of ventilation between cycles with high and between cycles with low magnitude of pendelluft during NAVA, PAV + and PSV. **Figure S1.** Assistance titration in NAVA, PAV + and PSV. **Figure S2.** Quasi-static driving airway pressure for respiratory system and lung in PAV + during the trial. **Figure S3**. Additional indices of respiratory drive (dPdi/dt and dPes/dt) in NAVA, PAV + and PSV. **Figure S4.** Dorsal fraction of ventilation in cycles with high compared with low magnitude of pendelluft in NAVA, PAV + and PSV at similar tidal volume. **Figure S5.** Dorsal fraction of ventilation in cycles with high compared with low magnitude of pendelluft in NAVA, PAV + and PSV at similar tidal volume. **Methods S1.** Ethics approval. **Methods S2.** Transition from controlled ventilation to partial ventilatory support. **Methods S3.** Study protocol. **Methods S4.** Quasi-static driving airway pressure for respiratory system and lung in PAV+ during the trial. **Methods S5.** Additional indices of respiratory drive (dPdi/dt and dPes/dt). Methods S6. Dorsal fraction of ventilation in ventilatory cycles with low and high magnitude of pendelluft

## Data Availability

The datasets and materials used and/or analyzed during the current study are available from the corresponding author on reasonable request.

## Abbreviations

ARDS: Acute respiratory distress syndrome
EIT: Electrical impedance tomography
NAVA: Neurally adjusted ventilatory assist
PaO2:FiO2: Ratio of arterial partial pressure of oxygen to inspired oxygen fraction
PAV +: Proportional assist ventilation plus
PBW: Predicted body weight
PCV: Pressure-controlled ventilation
PEEP: Positive end-expiratory pressure
PSV: Pressure support ventilation
P_0.1_: Airway occlusion pressure at 100 ms
P_aw_: Airway pressure
P_es_: Esophageal pressure
P_di_: Transdiaphragmatic pressure
P_g_: Gastric pressure
P_L_: Transpulmonary pressure
PTP_300ms_: Pressure–time product of the first 300 ms
PTP_min_: Pressure–time product per minute
RR: Respiratory rate
V_T_: Tidal volume
∆Pes _100 ms_: Delta esophageal swing of the first 100 ms
