# Supplementary material for: Pendelluft in hypoxemic patients resuming spontaneous breathing: proportional modes versus pressure support ventilation
Source: Ann Intensive Care. 2023 Dec 20;13:131. doi: 10.1186/s13613-023-01230-w (PMC10733241; doi:10.1186/s13613-023-01230-w)
Supplement: Supplementary file 1 — Additional file 1: Table S1. Demographic and clinical characteristics of the study population. Table S2. Patients’ data at the study entry and the individualized levels of PEEP and comparable levels of assistance in NAVA, PAV + and PSV during the trial. Table S3. Comparison of tidal volume, esophageal swing and pressure–time product per minute at the titration period between NAVA, PAV + and PSV. Table S4. Individual differences of the respiratory variables during crossover trial, using PS.as reference value. Table S5. Comparison of tidal volume and dorsal fraction of ventilation between cycles with high and between cycles with low magnitude of pendelluft during NAVA, PAV + and PSV. Figure S1. Assistance titration in NAVA, PAV + and PSV. Figure S2. Quasi-static driving airway pressure for respiratory system and lung in PAV + during the trial. Figure S3. Additional indices of respiratory drive (dPdi/dt and dPes/dt) in NAVA, PAV + and PSV. Figure S4. Dorsal fraction of ventilation in cycles with high compared with low magnitude of pendelluft in NAVA, PAV + and PSV at similar tidal volume. Figure S5. Dorsal fraction of ventilation in cycles with high compared with low magnitude of pendelluft in NAVA, PAV + and PSV at similar tidal volume. Methods S1. Ethics approval. Methods S2. Transition from controlled ventilation to partial ventilatory support. Methods S3. Study protocol. Methods S4. Quasi-static driving airway pressure for respiratory system and lung in PAV+ during the trial. Methods S5. Additional indices of respiratory drive (dPdi/dt and dPes/dt). Methods S6. Dorsal fraction of ventilation in ventilatory cycles with low and high magnitude of pendelluft [file 13613_2023_1230_MOESM1_ESM.pdf]

SUPPLEMENTARY MATERIAL

Additional file 1

Pendelluft in hypoxemic patients resuming spontaneous breathing: Proportional modes versus pressure support ventilation

Daniel H. Arellano, Roberto Brito, Caio C.A Morais, Pablo Ruiz-Rudolph, Abraham I.J. Gajardo, Dannette V. Guiñez, Marioli T. Lazo, Ivan Ramirez, Verónica A. Rojas, María A. Cerda, Juan N. Medel, Victor Illanes, Nivia R. Estuardo, Alejandro Bruhn, Laurent J. Brochard, Marcelo B. P. Amato, #Rodrigo A. Cornejo

#Corresponding author:

Rodrigo A. Cornejo MD, FACP. Unidad de Pacientes Críticos, Departamento de Medicina, Hospital Clínico Universidad de Chile. Dr. Carlos Lorca Tobar 999, Independencia, Santiago, Chile. e-mail: racornej@gmail.com. Postal code: 8380456

METHODS:

Methods S1. Ethics approval

Methods S2. Transition from controlled ventilation to partial ventilatory support

Methods S3. Study protocol

a. PEEP titration
b. Ventilatory settings in PSV, PAV+ and NAVA
c. Pendelluft magnitude assessment

Methods S4. Quasi-static driving airway pressure for respiratory system and lung in PAV+ during the trial

Methods S5. Additional indices of respiratory drive (dP_{di}/dt and dP_{es}/dt)

Methods S6. Dorsal fraction of ventilation in ventilatory cycles with low and high magnitude of pendelluft

TABLES

Table S1. Demographic and clinical characteristics of the study population

Table S2. Patients' data at the study entry and the individualized levels of PEEP and comparable levels of assistance in NAVA, PAV+ and PSV during the trial

Table S3. Comparison of tidal volume, esophageal swing and pressure-time product per minute at the titration period between NAVA, PAV+ and PSV

Table S4. Individual differences of the respiratory variables during crossover trial, using PS as reference value

Table S5. Comparison of tidal volume and dorsal fraction of ventilation between cycles with high and between cycles with low magnitude of pendelluft during NAVA, PAV+ and PSV

FIGURES

Figure S1. Assistance titration in NAVA, PAV+ and PSV

Figure S2. Quasi-static driving airway pressure for respiratory system and lung in PAV+ during the trial

Figure S3. Additional indices of respiratory drive (dP_{di}/dt and dP_{es}/dt) in NAVA, PAV+ and PSV.

Figure S4. Dorsal fraction of ventilation in cycles with high compared with low magnitude of pendelluft in NAVA, PAV+ and PSV at similar tidal volume

Figure S5. Dorsal fraction of ventilation in cycles with high compared with low magnitude of pendelluft in NAVA, PAV+ and PSV at similar tidal volume

METHODS

Methods S1. Ethics approval

This study represents the 2nd phase of the project FONDECYT Nº 1161510 entitled "Determinants of lung injury induced by spontaneous breathing efforts during the transition from controlled ventilation to partial ventilatory support in ARDS patients", which was approved by the Institutional Ethics Committee (approval number N.027/2016, Comité Ético Científico Hospital Clínico Universidad de Chile). Informed consent was obtained from the patients' next of kin.

Methods S2: Transition from controlled to partial ventilatory support

We included patients who presented moderate-severe ARDS in the early phase and received controlled ventilation for more than 72 hours, in whom the attending physician had decided the transition from controlled to partial ventilatory support 24 hours before the spontaneous study onset. The criteria commonly considered at the ICU for switching to partial ventilatory support in ARDS patients are: evidence for some reversal of the underlying cause for acute respiratory failure, PaO_2:FiO_2 ratio 150 mmHg or higher; to be in supine position, acceptable acid-base balance (arterial pH 7.25 or higher); blood pressure and perfusion supportable without requirement for significant vasopressor support (Norepinephrine ≤0.2 mcg/kg/min) and with lactic acid <4 mmol/L.

To initiate the transition from controlled ventilation and deep sedation, the infusion dose of sedative and opioid agents was reduced in steps to achieve moderate-light sedation (Richmond Agitation-Sedation Scale -2 to -3), initiating with a 50% decrease to ensure patient safety, and the ventilatory mode was changed from volume-controlled to assisted-

pressure controlled (PCV) mode favoring spontaneous breathing, or to BILEVEL mode after detection of a regular patient's respiratory rate ≥10 bpm. Henceforth, the infusion dose of sedative/opioid was adjusted according to clinical tolerance and RASS target. Thus, the patients maintained spontaneous breathing under assisted-PCV or BILEVEL until the spontaneous modes trial started with the phase of assistant titration.

Methods S3. Study protocol

a. PEEP titration

Twelve hours before the assessments, we assessed the quasi-static respiratory mechanics including chest wall elastance and defined an individualized level of PEEP according to EIT. First, a recruitment maneuver was performed, and then, a decremental PEEP-titration trial was accomplished once insured hemodynamic stability and deep sedation (without SB efforts) were achieved. Briefly, optimal PEEP was defined as the PEEP associated with the lowest combination of collapse and overdistension according to EIT monitoring. Technical details were recently published by Cornejo et al. (S1).

b. Ventilatory settings in PSV, PAV+ and NAVA

Two different ventilators were used: Maquet® Servo-I version 8.0 (for PSV and NAVA) and Medtronic® Puritan Bennett 840 (for PAV+). Flow-trigger sensitivity was set at 2 L/min, meanwhile, cycling-off was set at 25% of peak inspiratory flow in PSV and 3 L/min in PAV+. NAVA triggering sensitivity and cycling off were fixed at 0.5 μV and 70% of the electrical activity of the diaphragm (EA_{di}) peak, respectively.

To obtain the best level of assistance in each mode specific approaches were performed. For PSV, we randomly applied 0, 5, 10 and 15 cmH_2O of pressure support (2

minutes each). For NAVA, we randomly applied 0.5, 1.0 and 1.5 cmH_2O/μV (2 minutes each). For PAV+, we used the gain adjustment algorithm suggested by Carteaux et al. (S2). All with PSV mode as the reference. Individual values are shown in **Table S2** and assistance titration data in **Figure S1.** In addition, the comparisons in tidal volume, esophageal swing and pressure-time product per minute between NAVA, PAV+ and PSV at the assistance titration, are shown in **Table S3**.

To ensure intrapatient reproducibility, the patients were maintained at a semi-recumbent position (45º) throughout all study phases. Data were calculated from the last 15-minutes monitoring in each mode. Individual differences of the respiratory variables during crossover trial, using PSV as reference value, are shown in **Table S4**.

Inspiratory trigger delay in NAVA, PAV+ and PSV was analyzed to confirm if the delta esophageal swing of the first 100 ms ($\Delta P_{es\ 100ms}$) was a feasible proxy of $P_{0.1}$. The reported values of inspiratory trigger delay on bench studies for Servo-I and Puritan Bennett 840 range between <60 to >100 ms (depending of the inspiratory effort, the assistance level and the respiratory simulation system) (S3-S5). Nevertheless, in a recent study performed in hypoxemic patients, Schmidt et al. described an inspiratory trigger delay significantly higher than 100 ms during NAVA, PAV+ and PSV. The authors defined the inspiratory trigger delay as the time difference between the beginning of the increase in the EA_{di} signal and the beginning of the ventilator inspiratory flow (S6). We therefore analyzed the inspiratory trigger delay, using a similar definition that considered the beginning of the deflection in the P_{es} signal instead of EA_{di}, in a convenience sample of 10 ventilatory cycles by mode during the trial. As expected, we obtained slightly lower values of inspiratory trigger delay compared

with Schmidt et al findings, but in all patients over 100 ms (PSV 110 [103–117] ms, NAVA 110 [100–130] ms, PAV+113 [107–127] ms). Hence, the use $\Delta P_{es\ 100ms}$ to estimate respiratory drive seems to be reasonable.

c. Pendelluft magnitude assessment:

The pendelluft magnitude was based on a method recently published, which allows qualitative detection (through angle phase assessment) and quantitative measurement of the phenomenon using EIT (S7). The EIT representation of the lungs displays the regional distribution of V_T for a ventilatory cycle (ventilation map). The ventilation map is divided into four region-of-interest (ROI), each covering the two lungs and representing 25% of the ventrodorsal diameter [ROI 1–4]. Non-dependent (ventral) lung region corresponds to [ROI 1 + ROI 2] and dependent (dorsal) lung region corresponds to [ROI 3 + ROI 4]. Tidal regional ventilation in time from non-dependent and dependent regions is expressed from 0 to 100% in each region (panel A of Figure 1). In ventilatory cycles with pendelluft, non-dependent region loses volume at the early stage on inspiration while dependent region starts inflation, producing that the wave of the non-dependent region would lag behind the dependent region. The phase angle visualization (panel B in Figure 1) allows to evidence the lost volume from non-dependent region with concomitant gain volume in dependent region during inspiration and not just inflation dyssynchrony. Panel C illustrates the volume displacement between ventral and dorsal regions, which is calculated as the average difference between non-dependent and dependent volume (%) throughout the inspiration (solid blue line in panel C). To avoid an overestimation of pendelluft magnitude, the pendelluft quantification initiates when the average difference crosses “0”.

Methods S4. Quasi-static driving airway pressure for respiratory system and lung in PAV+ during the trial

To obtain quasi-static measurements of airway driving pressure (ΔP_{aw}) and transpulmonary driving pressure (ΔP_L) in PAV+ during the trial, we selected the cycles with inspiratory pauses in each patient. Quasi-static ΔPaw was calculated as the difference between plateau airway pressure and total PEEP. Quasi-static ΔP_L was calculated as the difference between [plateau airway pressure - plateau esophageal pressure] and [total PEEP - expiratory esophageal pressure]. **Figure S2** shows the averages of quasi-static ΔP_{aw} and ΔP_L by patient.

Methods S5. Additional indices of respiratory drive (dP_{di}/dt and dP_{es}/dt)

We calculated the rate of increase in transdiaphragmatic pressure (dPdi/dt) and the rate of increase in esophageal pressure swing (dPes/dt), as the change in Pdi and Pes during inspiration, respectively, divided by inspiratory time. We compared dP_{di}/dt and dP_{es}/dt between the three modes using repeated measures ANOVA followed by Dunnett‘s post-hoc test (A and B in the **Figure S3**), and their associations with pendelluft using mixed-effects models (See Table 2). Although dP_di/dt and dP_{es}/dt are validated indexes of respiratory drive, we selected $\Delta P_{es\ 100ms}$ as representative variable of respiratory drive, because diaphragmatic-derived indexes may underestimate this parameter in high-respiratory drive context (S8). On the other hand, a potential dissociation between respiratory drive and inspiratory effort (assessed by ΔP_{es}) may be observed in the presence of muscular weakness (S9**)**.

Methods S6. Dorsal fraction of ventilation

To provide the estimated increase of the regional tidal ventilation in the dependent lung regions from pendelluft in the different modes we performed the following tasks:

a) We obtained the histograms of pendelluft by patient and identified the patients who developed pendelluft of different magnitudes (between 10% to 25%), knowing that some patients only presented pendelluft of very low magnitude (<10%) or not presented pendelluft of high magnitude. Then, in each identified patient, we selected the ventilatory cycles with tidal volume of 6 - 9 ml/kg PBW, because spontaneous modes (especially proportional modes) promote a high variability of the respiratory pattern and regional ventilation themselves. The number of patients included in each mode was different in response to the aforementioned criteria. Fourteen patients satisfied the criteria in NAVA, twelve in PAV+ and 5 in PSV.

b) We divided the ventilatory cycles of these patients according to the magnitude of pendelluft between low (pendelluft 10-15%) and high (pendelluft 20-25%) magnitude in the three modes NAVA, PAV+ and PSV based on our previous data (S7).

c) We estimated the dorsal fraction of ventilation per ventilatory cycle as the ratio between tidal volume in the dependent region and total tidal volume ($V_{T\ dep}$ / V_T total) multiplicated by 100.

d) We compared the tidal volume and dorsal fraction of ventilation between ventilatory cycles with low and high magnitude of pendelluft in each spontaneous mode separately, using paired t-test (**Figure S4**).

d) We also compared the percentage of change (Δ%) in dorsal fraction of ventilation between cycles with high and low magnitude of pendelluft during NAVA, PAV+ and PSV (**Figure S5**), using one-way ANOVA test with Tukey post-hoc analysis.

e) We finally compared the tidal volume and dorsal fraction of ventilation between cycles with high and between cycles with low magnitude of pendelluft during NAVA, PAV+ and PSV, using one-way ANOVA test with Tukey post-hoc analysis (**Table S5**).

Table S1. Demographic and clinical characteristics of the study population

Pt (N)	Age (y.o)	Sex (M/F)	BMI (kg/m^2)	ARDS cause	CT Phenotype	SOFA (max)	$PaO_2:FiO_2$ (min)	ICU LoS (days)	MV duration (days)	In-Hospital Mortality
1	30	M	33	OLT/SIRS	Patchy	18	78	41	30	Yes
2	66	F	22	Pneumonia	Diffuse	17	132	46	46	Yes
3	75	M	23	Pneumonia	Lobar	11	90	35	*†36	No
4	67	M	23	Pneumonia	Diffuse	13	160	41	*†43	No
5	77	F	36	Sepsis	Patchy	16	134	40	†37	No
6	64	M	32	Sepsis	Lobar	13	77	10	9	No
7	49	F	21	Pneumonia	Patchy	12	149	33	11	No
8	31	M	23	Sepsis	Patchy	8	157	39	†30	No
9	61	F	32	Sepsis	Lobar	11	125	17	16	No
10	56	F	32	Pneumonia	Patchy	6	152	11	9	No
11	60	M	35	Pneumonia	Lobar	8	107	38	†36	No
12	63	F	31	Pneumonia	Patchy	13	92	9	8	No
13	57	M	19	Sepsis	Diffuse	10	176	7	7	No
14	52	M	29	Pneumonia	Patchy	14	98	13	10	No
15	63	M	33	Pneumonia	Lobar	9	103	9	7	No
16	73	M	28	Pneumonia	Patchy	6	120	12	10	No
17	54	M	30	Pneumonia	Lobar	11	84	27	†27	Yes
18	32	M	23	Pneumonia	Patchy	15	138	74	†74	Yes
19	47	M	35	Pneumonia	Diffuse	7	153	12	8	No
20	67	M	22	Aspiration	Patchy	19	80	49	†31	No

Pt: patient; M/F: male/female; BMI: body mass index; ARDS: acute respiratory distress syndrome; CT image: computed tomography findings; SOFA: maximum value of Sequential Organ Failure Assessment Score in the acute phase of ARDS; $PaO_2:FiO_2$: minimum value of the ratio of arterial oxygen partial pressure to fractional inspired oxygen in the acute phase of ARDS; ICU: intensive care unit; LoS: length of stay; MV duration: total duration of mechanical ventilation; OLT/SIRS: orthotopic liver transplant/systemic inflammatory response syndrome.

*These patients initiated their time on mechanical ventilation before the ICU admission

† These patients were tracheostomized

Table S2. Patients' data at the study entry and the individualized levels of PEEP and comparable levels of assistance in NAVA, PAV+ and PSV during the trial

**Pt** (N)	**MV time** (days)	$C_{RS\ before}$ (ml/cm H_2O)	$E_{L\ before}$ (cm H_2O/L)	**PEEP** (cm H_2O)	**NAVA** Assist (μV)	**PAV+** Assist (%)	**PSV** Assist (cm H_2O)
1	24	31	25.6	11	0.5	40	10
2	17	45	15.2	12	0.5	30	5
3	17	48	14.5	10	1.0	50	10
4	24	63	9.9	6	1.0	60	10
5	10	36	18.6	16	1.0	55	10
6	4	40	13.4	10	1.5	60	10
7	5	52	12.7	8	1.0	40	5
8	9	43	16.3	7	1.5	50	10
9	12	52	8.0	6	1.5	60	10
10	5	28	25.7	13	1.0	60	10
11	5	33	24.1	7	0.5	40	5
12	6	26	29.5	7	1.0	45	5
13	5	27	31.2	10	0.5	50	10
14	9	24	32.1	6	1.0	50	10
15	5	39	19.0	6	1.0	60	10
16	5	58	9.0	12	1.0	45	10
17	14	31	23.6	12	1.0	35	10
18	13	45	15.9	13	1.0	40	5
19	7	33	25.8	12	1.0	50	5
20	12	29	27.4	10	1.0	50	5

Pt: patient. MV: mechanical ventilation; $C_{RS\ before}$: quasi-static compliance of the respiratory system 12-hours before spontaneous modes study; $E_{L\ before}$: Lung elastance 12-hours before spontaneous modes study; PEEP: positive end-expiratory pressure (adjusted according to electrical impedance tomography). Assist: Individualized assistance during each ventilatory mode (see Study Protocol in the Online Data Supplement).

Table S3. Comparisons of tidal volume, esophageal pressure swing and pressure-time product per minute between NAVA, PAV+ and PSV at the assistance titration.

	NAVA	PAV+	PSV	p-value
V_T, ml/kg PBW, median [IQR]	7.7 [6.5 – 8.0]	7.2 [6.1 – 7.8]	7.2 [6.8 – 8.4]	0.1165
ΔP_{es}, cm H_2O, median [IQR]	-7.5 [-9.5 – -6.2]	-7.9 [-10 – -5.9]	-6.2 [-9.9 – -4.4]	0.0759
PTP_{min} cm H_2O s/min, median [IQR]	121 [86 – 154]	155 [117 – 186]	129 [63 – 157]	0.0759

Table S4. individual differences of the respiratory variables during crossover trial, using PS as reference value.

	NAVA	PAV+
ΔTidal volume, ml/kg PBW, median [IQR]	0.073 [-14.52 – 1.067]	-0.013 [-0.93 – 0.57]
ΔRespiratory rate, bpm, mean ± SD	2.6 ± 4.4	2.7 ± 5.5
ΔVolume minute ventilation, mean ± SD	1.2 ± 2.7	0.9 ± 3.5
ΔPeak P_{aw}, cm H_2O, mean ± SD	2.4 ± 4.4	-2.0 ± 2.8
Δ-ΔP_{es}, cm H_2O, mean ± SD	-1.2 ± 3.6	-3.7 ± 4.1
Δ-ΔP_L, cm H_2O, median [IQR]	3.18 [1.06 – 6.08]	2.01 [-0.83 – 3.92]
Δ-ΔP_{di}, cm H_2O, median [IQR]	0.86 [-1.29 – 2.47]	2.57 [1.09 – 5.79]
ΔPaO_2, mmHg, median [IQR]	-2.1 [-8.5 – 1.9]	0.1 [-8 – 13.6]
ΔPaO_2:FiO_2, mean ± SD	-8 ± 43	-0.8 ± 49
$\Delta PaCO_2$, mmHg, mean ± SD	-0.28 ± 1.94	-0.005 ± 2.07
ΔpH, median [IQR]	0 [-0.01 – 0.01]	-0.005 [-0.02 – 0.01]
ΔVentilatory ratio, mean ± SD	0.3 ± 0.5	0.2. ± 0.5

Note: compared with PSV, peak Paw was higher in NAVA and lower in PAV+. The higher peak P_{aw} in NAVA may be explained by the high variability in EA_{di} signal (**S10**), while the lower peak P_{aw} in PAV+ seems to be related with a low-moderate gain support in PAV+ (**S11,S12**).

Table S4. **Comparison of tidal volume and dorsal fraction of ventilation in cycles with high and low magnitude of pendelluft during NAVA, PAV+ and PSV**

	**NAVA**	**PAV+**	**PSV**	**p-value**
Tidal volume, ml/kg PBW, median [IQR]				
Cycles with low pendelluft	7.6 ± 1.1	7.3 ± 0.9*	8.0 ± 0.9	0.0030
Cycles with high pendelluft	7.5 ± 1.0	7.2 ± 0.8*	7.9 ± 0.9	0.0365
Dorsal fraction of ventilation (%)				
Cycles with low pendelluft	42 ± 12	52 ± 13*#	39 ± 8	<0.0001
Cycles with high pendelluft	44 ± 12	57 ± 13*#	42 ± 8	<0.0001

p-value of one-way ANOVA test with Tukey post-hoc analysis
* p< 0.05 between PAV+ and PSV
# p< 0.05 between PAV+ and NAVA

Figure S1. Assistance titration in NAVA, PAV+ and PSV

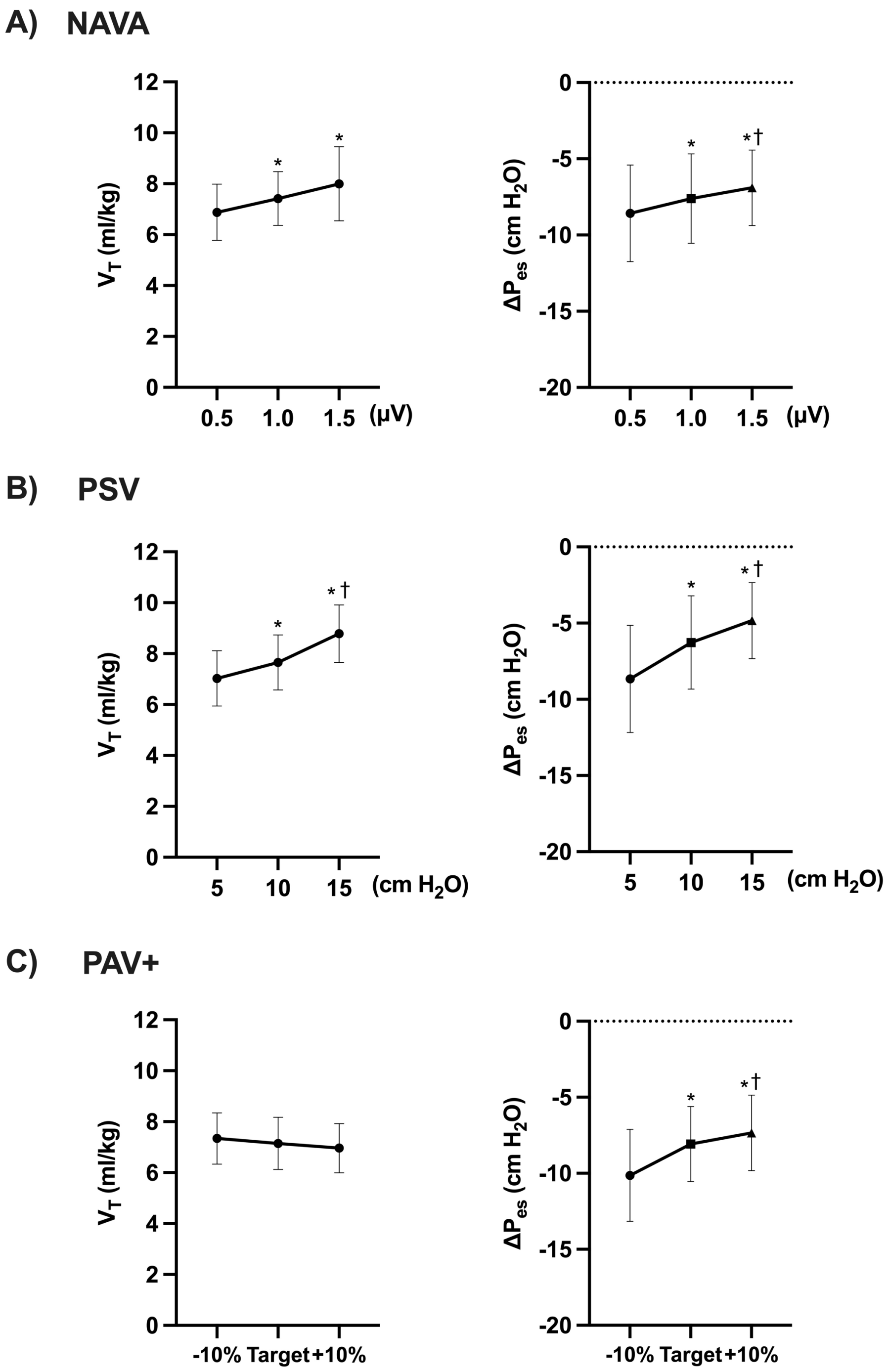

Figure S1 shows the progress of V_T and ΔP_{es} during NAVA (A), PSV (B) and PAV+ (C)) at three different levels of assistance in ascending scale: low, intermediate and high level of assistance (i.e. for NAVA 0.5 – 1.0 – 1.5 µV and for PSV 5 – 10 – 15 cm H_20). In the case

of PAV+, we graphed the optimal level of assistance and 10% lower and 10% higher with respect to the target. Note that when the assistance increased, ΔP_{es} significantly decreased in the three modes. Otherwise, V_T significantly increased in PSV and NAVA, but not in PAV+

*$p<0.05$ compared with low level of assistance

†$p<0.05$ compared with intermediate or target level of assistance

Figure S2. Quasi-static driving airway pressure for respiratory system and lung in PAV+ during the trial

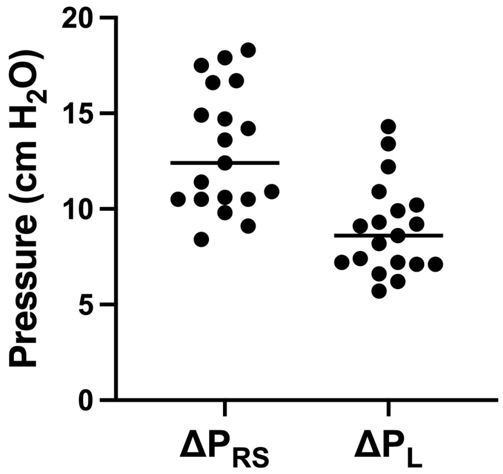

Figure S2 shows the values of individual average and median of quasi-static driving airway pressure for respiratory system (ΔP_{RS}) and lung (ΔP_L) in PAV+ during the trial.

Figure S3. Additional indices of respiratory drive (dP_{di}/dt and dP_{es}/dt)

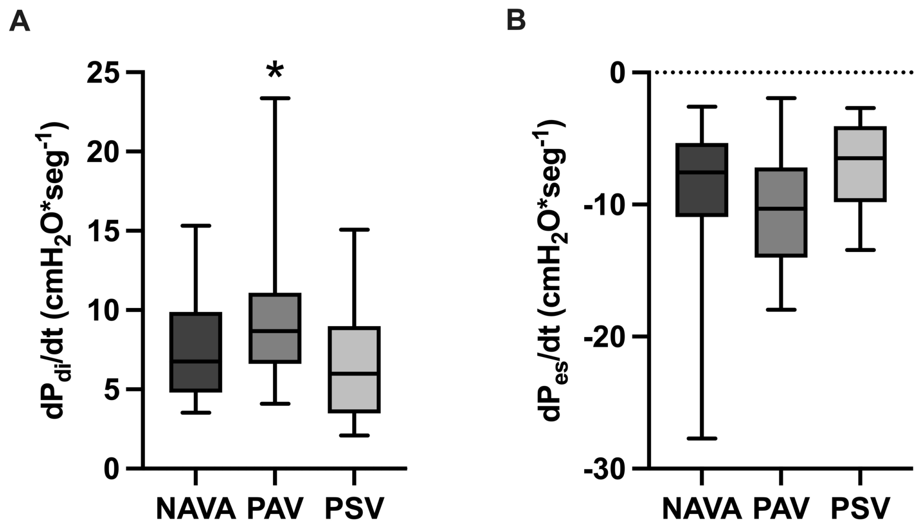

dP_{di}/dt corresponds to the rate of increase in transdiaphragmatic pressure during inspiration divided by the inspiratory time. dP_{es}/dt corresponds to the rate of increase in esophageal pressure swing during inspiration divided by the inspiratory time.

*$p<0.05$ compared with PSV

Figure S4. Dorsal fraction of ventilation in cycles with high and low magnitude of pendelluft during NAVA, PAV+ and PSV

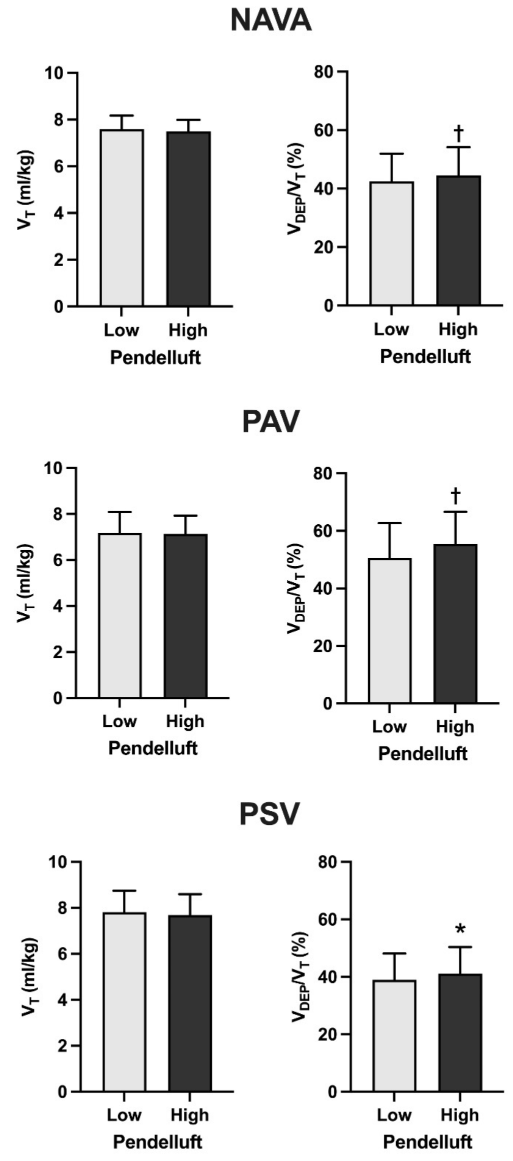

In each spontaneous mode (NAVA, PAV+ and PSV), we compared the tidal volume (V_T) and dorsal fraction of ventilation ([$V_{T\,dep}$ / V_T total] multiplicated by 100) in cycles with low (soft gray) and high (dark gray) magnitude of pendelluft from the identified patients.

*$p < 0,05$, $^{\dagger}p < 0,001$, $^{\ddagger}p < 0,0001$

Figure S5. Percentage of change (Δ%) in dorsal fraction of ventilation between cycles with high and low magnitude of pendelluft during NAVA, PAV+ and PSV

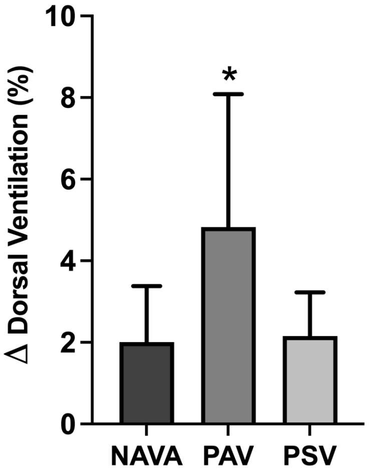

*p<0.05 compared with PSV and NAVA

REFERENCES

S1 Cornejo R, Iturrieta P, Olegário TMM, Kajiyama C, Arellano D, Guiñez D, et al. Estimation of changes in cyclic lung strain by electrical impedance tomography: Proof-of-concept study. Acta Anaesthesiol Scand 2021;65:228-235.

S2 Carteaux G, Mancebo J, Mercat A, Dellamonica J, Richard JCM, Aguirre-Bermeo H, et al. Bedside adjustment of proportional assist ventilation to target a predefined range of respiratory effort. Crit Care Med 2013;4:2125–2132.

S3 Thille AW, Lyazidi A, Richard JC, Galia F, Brochard L. A bench study of intensive-care-unit ventilators: new versus old and turbine-based versus compressed gas-based ventilators. Intensive Care Med. 2009; 35: 1368-76.

S4 Sassoon CSh. Triggering of the ventilator in patient-ventilator interactions. Respir Care. 2011 Jan;56(1):39-51

S5 Terado M, Ichiba S, Nagano O, Ujike Y. Evaluation of pressure support ventilation with seven different ventilators using Active Servo Lung 5000. Acta Med Okayama. 2008; 62: 127-33

S6 Schmidt M, Kindler F, Cecchini J, Poitou T, Morawiec E, Persichini R, et al. Neurally adjusted ventilatory assist and proportional assist ventilation both improve patient-ventilator interaction. Crit Care. 2015; 19: 56

S7 Cornejo RA, Arellano DH, Ruiz-Rudolph P, Guiñez DV, Morais CCA, Gajardo AIJ, et al. Inflammatory biomarkers and pendelluft magnitude in ards patients transitioning from controlled to partial support ventilation. Sci Reports 2022;12:20233.

S8 Vaporidi K, Akoumianaki E, Telias I, Goligher EC, Brochard L, Georgopoulos D. Respiratory Drive in Critically Ill Patients. Pathophysiology and Clinical Implications. Am J Respir Crit Care Med. 2020; 201: 20-32

S9 Spinelli E, Mauri T, Beitler JR, Pesenti A, Brodie D. Respiratory drive in the acute respiratory distress syndrome: pathophysiology, monitoring, and therapeutic interventions. Intensive Care Med. 2020; 46: 606-618.

S10 Diniz-Silva F, Moriya HT, Alencar AM, Amato MBP, Carvalho CRR, Ferreira JC. Neurally adjusted ventilatory assist vs. pressure support to deliver protective mechanical ventilation in patients with acute respiratory distress syndrome: a randomized crossover trial. Ann Intensive Care. 2020; 10: 18.

S11 Botha J, Green C, Carney I, Haji K, Gupta S, Tiruvoipati R. Proportional assist ventilation versus pressure support ventilation in weaning ventilation: a pilot randomised controlled trial. Crit Care Resusc. 2018; 20: 33-40.

S12 Spieth PM, Güldner A, Beda A, Carvalho N, Nowack T, Krause A, Rentzsch I, Suchantke S, Thal SC, Engelhard K, Kasper M, Koch T, Pelosi P, de Abreu MG. Comparative effects of proportional assist and variable pressure support ventilation on lung function and damage in experimental lung injury. Crit Care Med. 2012; 40: 2654-61
